# Effects of a Nurse-Led, Acceptance and Commitment–Based Palliative Intervention on the Psychospiritual Well-Being of Patients With Progressive Neurological Diseases: Protocol for a Mixed Methods Study

**DOI:** 10.2196/105727

**Published:** 2026-08-13

**Authors:** Lily Man Lee Chan, Wendy Wing Tak Lam, Koon Ho Chan, Shirley Yin Yu Pang, Man Auyeung, Jojo Yan Yan Kwok

**Affiliations:** 1 School of Nursing, Li Ka Shing Faculty of Medicine, University of Hong Kong, Room 558, 5/F, HKUMed Academic Building, 3 Sassoon Road, Pokfulam, Hong Kong, China (Hong Kong), (852) 39176644; 2 Centre for Psycho-Oncological Research and Training, Division of Behavioural Sciences, School of Public Health, University of Hong Kong, Hong Kong, China (Hong Kong); 3 Jockey Club Institute of Cancer Care, Li Ka Shing Faculty of Medicine, University of Hong Kong, Hong Kong, China (Hong Kong); 4 Department of Medicine, School of Clinical Medicine, Li Ka Shing Faculty of Medicine, University of Hong Kong, Hong Kong, China (Hong Kong); 5 Neuroimmunology and Neuroinflammation Research Laboratory, Li Ka Shing Faculty of Medicine, University of Hong Kong, Hong Kong, China (Hong Kong); 6 Research Center of Heart, Brain, Hormone and Healthy Aging, Li Ka Shing Faculty of Medicine, University of Hong Kong, Hong Kong, China (Hong Kong); 7 Department of Medicine, Queen Mary Hospital, Hong Kong, China (Hong Kong); 8 Department of Medicine, Pamela Youde Nethersole Eastern Hospital, Hong Kong, China (Hong Kong); 9 Center on Behavioral Health, Faculty of Social Sciences, University of Hong Kong, Hong Kong, China (Hong Kong)

**Keywords:** progressive neurological diseases, acceptance and commitment therapy, palliative care, supportive-expressive therapy, psychological distress, spiritual well-being, meaning in life, death anxiety, psychological flexibility, illness perception

## Abstract

**Background:**

Progressive neurological diseases (PNDs) such as Parkinson disease and multiple sclerosis cause profound motor and nonmotor symptom burdens that significantly impair health-related quality of life. Beyond physical challenges, patients face profound psychospiritual distress driven by prognostic uncertainty, loss of autonomy, and existential concerns. While palliative care can address these multidimensional needs, it remains underused in neurological populations, where conventional care predominantly focuses on motor symptom management.

**Objective:**

This study aims to evaluate the effects and acceptability of a nurse-led, acceptance and commitment–based palliative program on psychological distress, psychospiritual well-being, and related health outcomes among individuals living with PNDs.

**Methods:**

This is a 2-arm, parallel-group randomized controlled trial using an explanatory sequential mixed methods design. A total of 138 participants with PNDs will be recruited and randomized 1:1 to either the intervention group (n=69) or a befriending control group (n=69). The intervention consists of five 60- to 90-minute individual sessions delivered over 3 months by trained nurses integrating acceptance and commitment therapy–based strategies (eg, mindfulness, cognitive defusion, and value-guided action) with supportive-expressive approaches. The control group receives five 30- to 60-minute active befriending sessions. The primary outcome is psychological distress, assessed via the Hospital Anxiety and Depression Scale. Secondary outcomes include spiritual well-being, meaning in life, psychological flexibility, illness perception, palliative care needs, health-related quality of life, and death anxiety. Assessments will be conducted at baseline (T0), 1 week after the intervention (T1), and 3 months after the intervention (T2). Quantitative data will be analyzed using linear mixed-effects models under the intention-to-treat principle. Following the intervention, a purposive subsample will undergo semistructured qualitative interviews to explore intervention acceptability and participant experiences.

**Results:**

This trial obtained ethics approval from the Institutional Review Board of the University of Hong Kong and was funded by the Nethersole Institute of Continuing Holistic Health Education research grant 2022-2023 and the Health and Medical Research Fund (2022), Health Bureau. Recruitment commenced in September 2023. As of manuscript submission, 132 participants have been enrolled. Data analysis has not yet commenced. The results are expected to be published in late 2026.

**Conclusions:**

This study will provide empirical evidence regarding the effectiveness and acceptability of a nurse-led, acceptance and commitment–based palliative program tailored to the complex psychospiritual trajectories of patients with PNDs. If effective, this trial will offer a scalable model for integrating structured psychotherapeutic interventions within neurology, palliative care, and community nursing practices.

## Introduction

### Background

Progressive neurological diseases (PNDs), including Parkinson disease (PD) and multiple sclerosis (MS), affect more than 8 million people worldwide [1]. These conditions are characterized by mobility dysfunctions (eg, focal weakness, rigidity, spasticity, tremors, and coordination difficulties) and nonmotor symptoms (eg, pain, fatigue, urinary dysfunction, and cognitive impairment) [2,3]. The unpredictable disease trajectory, progressive functional loss, and shifting life roles contribute to substantial psychological and spiritual distress among individuals with PNDs [3,4]. Approximately 35% to 50% of individuals with PNDs experience anxiety and depression, which are associated with impaired quality of life (QoL), accelerated disease progression, frequent relapses, and increased care dependency [4]. Furthermore, spiritual distress is highly prevalent, arising from difficulties in sustaining a sense of meaning and purpose in life while confronting existential uncertainty, functional decline, and mortality [5,6].

Despite the multifaceted nature of PNDs, conventional clinical care focuses primarily on motor symptom management within a biomedical-oriented framework, overlooking nonmotor symptoms and psychospiritual needs [2,7]. Palliative care is a specialized form of care aimed at improving QoL for patients with life-limiting illness and their families through comprehensive assessments and management of physical, psychological, social, and spiritual needs, as well as care coordination and caregiver support [8]. While international guidelines advocate for early, person-centered palliative care integration within neurology, these services remain severely underused and are frequently restricted to end-of-life care stages [7,9].

A recent systematic review and meta-analysis of 15 trials (n=3431) demonstrated that palliative care interventions significantly improved symptom burden and satisfaction with care among individuals with PNDs [10]. However, their effects on QoL remain inconclusive, and most studies have focused on advanced disease stages. Existing trials often insufficiently address psychospiritual components despite their critical role in enhancing QoL. PNDs such as MS and PD are marked by fluctuating and unpredictable trajectories characterized by sporadic exacerbations, periods of recovery, and gradual decline [11]. These complexities make conventional prognosis-based palliative care models less suitable [12] and underscore the need for early, tailored, and integrated palliative approaches that address psychospiritual distress alongside physical symptoms [13].

Chronic illnesses such as PNDs can be conceptualized as a series of disruptive events—diagnosis, symptom progression, relapses, and end-of-life transitions—that challenge individuals’ values, identity, and coping capacity [14]. Prolonged disruption may hinder psychological adjustment, underscoring the need for palliative interventions that address psychospiritual distress alongside physical symptoms [6]. The Common-Sense Model of Self-Regulation (CSM) by Leventhal et al [15] explains how individuals form illness perceptions—such as beliefs about identity (disease label and symptoms), timeline (perceived duration), causes (underlying factors), consequences (perceived impacts of disease on everyday life), and control (the sense of agency in managing the disease)—that shape coping and outcomes. Additional dimensions include coherence (understanding of the illness) and emotional representations (emotional impact). Individuals with PNDs often perceive their condition as chronic, unpredictable, and uncontrollable, which can intensify helplessness and distress [16,17]. The prolonged disease duration, unpredictability of disease progression, and lack of definitive causes or cures further diminish one’s sense of control. Negative illness perceptions are associated with psychological distress, maladaptive coping, and poor treatment adherence [18,19], although coping strategies and social support may moderate these effects. Interventions that promote more adaptive illness representations and a greater sense of agency may therefore enhance coping and improve health outcomes [19,20].

Managing Cancer and Living Meaningfully (CALM) therapy is a brief, semistructured, and individualized psychotherapeutic intervention designed to alleviate distress and enhance psychological growth in patients with advanced cancer [21]. It focuses on four broad, interrelated domains in the context of living meaningfully with a life-threatening illness: (1) symptom management and communication with health care providers; (2) changes in the self and relations with close others; (3) spiritual well-being and sense of meaning and purpose; and (4) preparing for the future, sustaining hope, and facing mortality. Delivered over 3 to 6 therapist-led sessions, CALM uses a supportive-expressive approach to foster reflection, cognitive reframing, and meaning finding, facilitating mentalization—an ability to reflect on feeling states, differentiate from literal facts, and accept the possibility of multiple perspectives—which is crucial for patients to sustain hope and engage in disease planning and a meaningful life despite their illness. Empirical evidence demonstrates CALM’s effectiveness in reducing depressive symptoms, enhancing end-of-life preparedness, and improving overall QoL in patients with cancer [21]. Participants described CALM as a “safe place” for them to be “seen as a whole person” and that supports them to live meaningfully and “handle death issues in a peaceful way.” However, adaptation is required for individuals with PNDs, whose illness trajectories are prolonged, fluctuating, and marked by recurring losses. While CALM addresses existential concerns, many aspects of PNDs remain uncontrollable, and persistent uncertainty may intensify helplessness. Attempts to suppress distressing thoughts may paradoxically intensify psychological distress [22]. Thus, coping in PNDs requires not only meaning-centered exploration but also acceptance of ongoing uncertainty and decline.

Acceptance and commitment therapy (ACT), a third-wave cognitive behavioral therapy, conceptualizes psychological distress as arising from attempts to avoid or control unwanted internal experiences, which in turn restricts engagement in meaningful activities [22]. ACT aims to enhance psychological flexibility—the ability to accept internal experiences while pursuing value-based action—through 6 psychological processes: defusion, acceptance, contact with the present moment, the self as context, values, and committed actions [22]. Using experiential exercises, mindfulness, and metaphors, ACT helps individuals relate differently to distressing thoughts and emotions [22]. Acceptance- and mindfulness-based interventions have shown promise in promoting emotional balance and mental clarity through the cultivation of nonjudgmental awareness [23]. ACT has demonstrated effectiveness across chronic conditions, including chronic pain and diabetes, as well as common mental disorders [24]. Emerging evidence in palliative populations is also encouraging: a systematic review of 6 studies involving 261 patients with advanced cancer reported improvements in symptoms, psychological distress, and QoL [24]. Higher levels of acceptance are associated with more adaptive coping and fewer psychological morbidities and avoidance behaviors, supporting psychological flexibility as a protective factor in the face of serious illness [25].

Building on the empirical support for CALM therapy and ACT, we propose integrating these approaches to address the psychospiritual needs of individuals with PNDs. CALM therapy provides a structured framework to explore practical and existential concerns—such as symptom management, communication, and prognostic uncertainty—while fostering meaning and hope in the context of serious illness. However, many aspects of PNDs, including progressive decline and mortality, remain beyond personal control, and patients may become entangled in distressing thoughts and emotions that restrict engagement in meaningful activities. ACT complements this framework by cultivating acceptance and psychological flexibility, enabling patients to relate differently to difficult internal experiences and continue pursuing value-based living despite uncertainty and loss. Integrating these approaches may therefore help individuals with PNDs navigate distress, sustain meaning, and adapt more flexibly to disease progression.

Accordingly, this study aims to develop and evaluate a novel, nurse-led palliative intervention that integrates acceptance and commitment–based strategies within a supportive-expressive framework. The primary objective is to alleviate psychological distress; cultivate psychological flexibility; and optimize the multidimensional, psychospiritual well-being of individuals living with PNDs.

### Aims and Hypotheses

This study aims to (1) examine the effects of a nurse-led, acceptance and commitment–based, supportive-expressive palliative care program on anxiety and depressive symptoms (primary outcomes) and spiritual well-being, meaning in life, palliative care needs, psychological flexibility, illness perception, health-related QoL, and death anxiety (secondary outcomes) among individuals with PNDs compared to a befriending control group; and (2) explore participants’ perceptions and experience of the palliative program, specifically identifying its perceived utility, mechanisms of change (how and why it works or fails to work), and contextual factors influencing intervention acceptability, motivation, and real-life practice.

It is hypothesized that individuals with PNDs who receive the palliative program will demonstrate significantly greater improvement in psychological distress at the postintervention follow-up (T1) and 3 months after the intervention (T2) compared with those receiving the befriending control. In addition, it is hypothesized that participants in the palliative program will show greater positive changes in secondary psychological, spiritual, and palliative outcomes relative to the control across the same time points.

## Methods

### Study Design, Setting, and Sampling

This sequential mixed methods study comprises a 2-arm randomized controlled trial followed by a qualitative study. This protocol adheres to the SPIRIT (Standard Protocol Items: Recommendations for Interventional Trials) checklist [26]. Adults with PNDs will be recruited from outpatient neurology clinics (Queen Mary Hospital and Tung Wah Hospital) and patient support groups (Hong Kong Parkinson’s Disease Foundation and Hong Kong Neuro-Muscular Disease Association) in Hong Kong.

Eligible participants are adults (≥18 years) diagnosed with PNDs (eg, PD, MS, motor neuron disease, multiple system atrophy, progressive supranuclear palsy, muscular dystrophy, spinocerebellar ataxia, or spinal muscular atrophy) with psychological distress indicated by a Hospital Anxiety and Depression Scale score of 8 or higher on either the depression or anxiety subscale (sensitivity and specificity of 0.75‐0.89) [27] and able to communicate in Cantonese.

Exclusion criteria are severe cognitive impairment (Abbreviated Mental Test <6) [28], recent psychotherapy (within 6 months), or severe psychiatric or medical comorbidities limiting participation.

Power analysis using G*Power was performed to estimate the required sample size. Previous studies of mindfulness- and acceptance-based interventions have reported large effect sizes (0.96‐1.14) in people with MS [29] and moderate to large effects for anxiety (*d*=0.58) and depression (*d*=0.74) in PD [30]. Given the use of an active control (befriending), a conservative moderate effect size of 0.58 was assumed for the primary outcome (anxiety and depressive symptoms). Using a Bonferroni-adjusted significance level of 2.5% and 80% power, 118 participants (59 per arm) are required. Allowing for 15% attrition, the final target sample size is 138 participants (69 per arm).

For the subsequent qualitative phase, approximately 30 participants from the intervention group will be invited for postprogram semistructured interviews, with the final sample size guided by data saturation. Purposive maximum variation sampling (eg, gender, age, and educational level) will be used to capture diverse experiences. Additional participants will be recruited until no new themes emerge.

### Study Interventions

#### Intervention Group: Nurse-Led, Acceptance and Commitment–Based, Supportive-Expressive Palliative Program

The intervention comprises 5 individual 60- to 90-minute sessions delivered over 3 months. The intervention is semistructured, with the intervener trained to facilitate discussions on 5 key themes adapted from CALM therapy and informed by findings from a previous cross-sectional study and qualitative interviews exploring the illness experience and care needs of individuals with PNDs in Hong Kong [31,32]. These themes include (1) symptom control and communication with health care professionals, (2) changes in sense of self amid progressive loss of function, (3) social support and relationships with family and friends, (4) spiritual support and finding meaning and life purpose in the context of illness, and (5) fears about disease progression and planning for the uncertain future and mortality. The time devoted to each theme and the sequence in which they are addressed will depend on the salience of the patients’ concerns during the sessions. The intervention aims to encourage patients to express concerns related to their diagnosis and treatment, describe their lived experiences and emotions, and identify current challenges. On the other hand, the intervention seeks to cultivate acceptance and promote value-driven behaviors, enhancing patients’ psychological flexibility in the face of illness, thereby enhancing psychospiritual well-being. The intervener will use acceptance and commitment–based techniques such as experiential exercises, mindfulness, and metaphors to facilitate the process. The intervention outline is shown in Table 1. The intervention will be delivered by registered nurses who have received standardized training in ACT and CALM therapy and will follow a structured intervention manual. Homework exercises will be provided to encourage participants to practice skills between sessions and maintain a record of their progress. A workbook with session content, along with audio instructions for mindfulness exercises and defusion and acceptance strategies, will be provided for home practice. In each session, participants will be encouraged to share their experiences and discuss any difficulties encountered during practice to consolidate learning and troubleshoot challenges.

**Table 1.** An outline of the acceptance and commitment–based components with associated metaphors, experiential exercises, and home practice.

Session and main focus	Metaphors and/or experiential exercise	Home practice
Session 1		
Discuss the inevitability of emotional pain and how the mind can amplify psychological distress	Metaphors: “Mind is a ‘double-edged sword,’ / ‘problem-solving machine,’/ radio ‘doom and gloom’”	Coping strategies diary
Explore the emotional control agenda and consequences of trying to avoid unwanted thoughts and feelings	Coping strategies worksheet	—^a^
Introduce mindfulness as an alternative to cope with unwanted thoughts and feelings	Mindfulness exercise: “Acceptance techniques: Observe / breathe / expand / allow / expand awareness”	Diary of mindfulness practice: “Acceptance techniques”
Session 2		
Practice being present in the moment and experience thoughts and feelings in a nonjudgmental way	Mindful eating and body scan exercise	Diary of mindfulness practice: “Mindful body scan”
Discuss the consequences of struggling with or avoiding thoughts and feelings	Choice point diagram	—
Identify personal values and review whether behaviors align with these values	—	The Bull’s Eye values worksheet
Session 3		
Explore the problem of fusion with thoughts and feelings	Metaphor: “Hands as thoughts and feelings”	—
Practice defusion skills to step back and detach from difficult thoughts, feelings, or memories	Metaphor: “Emotional storm”; defusion exercise: “Dropping anchor”	Diary of defusion exercise: “Dropping anchor”
Notice how thoughts, feelings, and sensations get in the way of the things that are important in life	—	Physicalizing thoughts worksheet
Session 4		
Practice skills to notice the distinction between the self and thoughts, feelings, and sensations	Metaphor: “The sky and the weather”; defusion exercise: “The floating leaves on a moving stream”	Diary of defusion exercise: “The floating leaves on a moving stream”
Set value-driven, meaningful actions and realistic goals	Worksheet: “SMART goal setting”	Values and action card
Session 5		
Adopt an observer perspective on thoughts, feelings, and sensations	Metaphor: “The stage show”	—
Explore barriers to committed actions and strategies to overcome them	Worksheet: “What’s stopping you?”	—
Review mindfulness, defusion skills, metaphors, and exercises covered in previous sessions	—	—

a Not applicable.

The intervention aligns with recommended CALM therapy dosage (3 to 6 sessions over 3 to 6 months), with extended session length (60‐90 minutes) to incorporate ACT components. Evidence supports the feasibility and effectiveness of brief ACT-based interventions in palliative populations, with high adherence and promising effects on psychological distress [33]. Brief programs of no more than 5 sessions are associated with higher adherence, enhanced motivation, and better skill consolidation [24]. All sessions will be conducted in a private room at an academic institution or at the patients’ homes depending on their preference.

#### Control Group: Befriending Activity

Participants in the control group will receive 5 individual 30- to 60-minute befriending sessions over 3 months delivered by a trained registered nurse following the *Befriending Manual* [34]. Befriending serves as an active control condition designed to control for nonspecific therapeutic factors, including clinician contact time, attention and interpersonal support (eg, warmth and empathy), participant expectations of benefit, and the therapeutic alliance. Sessions focus on neutral, everyday topics of interest (eg, hobbies, pets, and current affairs) and avoid emotional exploration, problem-solving, or therapeutic techniques. The nurse will maintain a warm, friendly, and supportive stance throughout. For pragmatic reasons, extending unstructured befriending sessions beyond 60 minutes may lead to forced or unnatural conversations, which could inadvertently increase participant fatigue or lower retention rates.

### Outcome Evaluation

Outcomes will be assessed at baseline (T0), 1 week after the intervention (T1), and 3 months after the intervention (T2).

#### Primary Outcome

The Hospital Anxiety and Depression Scale (Chinese-Cantonese version) will be used to measure psychological distress. It is a 14-item self-report scale assessing anxiety and depression validated in neurological populations (Cronbach α=0.86) [27]. A higher score, rated on a 4-point Likert scale, indicates more severe stress [35]. Subscale scores ranging from 0 to 7 indicate “noncases,” scores from 8 to 10 suggest “possible cases,” and scores from 11 to 21 represent “probable cases” of anxiety or depression [36].

#### Secondary Outcomes

The Functional Assessment of Chronic Illness Therapy–Spiritual Well-Being 12-item scale (Chinese version) will be used to measure spiritual well-being. It assesses 3 constructs: belief, meaning, and sense of peace. All items are rated on a 5-point Likert scale (0=“not at all”; 4=“very much”). High scores indicate high levels of spiritual well-being (Cronbach α=0.711‐0.920 [37]).

The Meaning in Life Questionnaire (Chinese version) will be used to measure meaning in life [38]. It comprises 2 subscales: the presence of meaning in life and an individual’s drive and orientation toward finding meaning in life. It is rated on a 7-point Likert scale ranging from 1 (“absolutely untrue”) to 7 (“absolutely true”). Higher scores indicate higher degree of presence of meaning in life or search for meaning in life (Cronbach α=0.75‐0.85).

The Palliative Care Outcome Scale (Chinese version) will be used to assess symptom burden [39]. It comprises 10 items assessing physical symptoms, patient and family anxiety and well-being, information needs, and practical needs. It is scored on a 5-point Likert scale ranging from 0 (“not at all”) to 4 (“overwhelmingly”), with a total score of 40. It has shown good construct validity and test-retest reliability and acceptable internal consistency.

The Comprehensive Assessment of Acceptance and Commitment Therapy Processes (Chinese) will be used to measure psychological flexibility [40]. This 18-item scale consists of 3 subscales: openness to experience, behavioral awareness, and valued action. Each item is scored on a 5-point Likert scale from 1 (“strongly disagree”) to 5 (“strongly agree”). High scores indicate great psychological flexibility (Cronbach α=0.87).

The EQ-5D-5L (Chinese version) will be used to measure health-related QoL [41]. It includes 5 dimensions (mobility, self-care, usual activities, pain and discomfort, and anxiety and depression), each rated on 5 levels, and a visual analog scale (0‐100) measuring perceived overall health. Each dimension is scored on a 5-point response level ranging from “no problem” (level 1) to “extreme problem” (level 5). Health states are converted into a utility score (EQ-5D-5L index) using the Hong Kong value set [42], where 1 indicates full health and negative values indicate states worse than death.

The Brief Illness Perception Questionnaire (Chinese version) will be used to measure illness perception [43]. The 9-item scale evaluates cognitive and emotional representations of illness across domains such as consequences, timeline, control, identity, concerns, emotions, and coherence rated on scales from 0 to 10, with 1 open-ended causal item. Higher scores indicate more negative perceptions. However, higher scores on the personal control, treatment control, and coherence items reflect more positive perceptions and will be reverse scored when computing the total illness perception score. The instrument demonstrates acceptable reliability and validity and has been validated in palliative populations [43,44].

The Death and Dying Distress Scale (Chinese) will be used to assess distress about death and dying. This 15-item scale addresses fears about death and dying, the feeling of being a burden to others, and wasted opportunities [45]. It is rated on a 6-point Likert scale from 0 (“no distress”) to 5 (“very much distress”; Cronbach α=0.939).

### Baseline and Process Evaluation Measures

At baseline, sociodemographic and clinical data will be collected via self-report, including age, gender, marital status, educational level, religiosity, living status (eg, living alone, living with a spouse, living with family members, or other arrangements), financial assistance, disease onset, stage, comorbidities, and history of rehabilitation service use. Cognitive status will be screened using the Montreal Cognitive Assessment, Hong Kong version, a validated tool for Chinese patients with neurological conditions assessing executive function, attention, and verbal fluency [46]. Intervention expectancy and satisfaction will be measured using an adapted credibility and expectancy questionnaire [47]. Participants will rate the intervention’s logicality, usefulness, helpfulness, and perceived and expected improvement, as well as confidence in recommending it to others. The original English version of the credibility and expectancy questionnaire demonstrates satisfactory internal consistency (Cronbach α=0.79-0.90).

Following quantitative data collection, a subsample of participants will undergo individual semistructured interviews to explore perceived effects and acceptability of the intervention. Interviews will be conducted at home or in academic settings, audio recorded, and guided by open-ended questions.

### Data Collection Procedure

**Figure 1.**
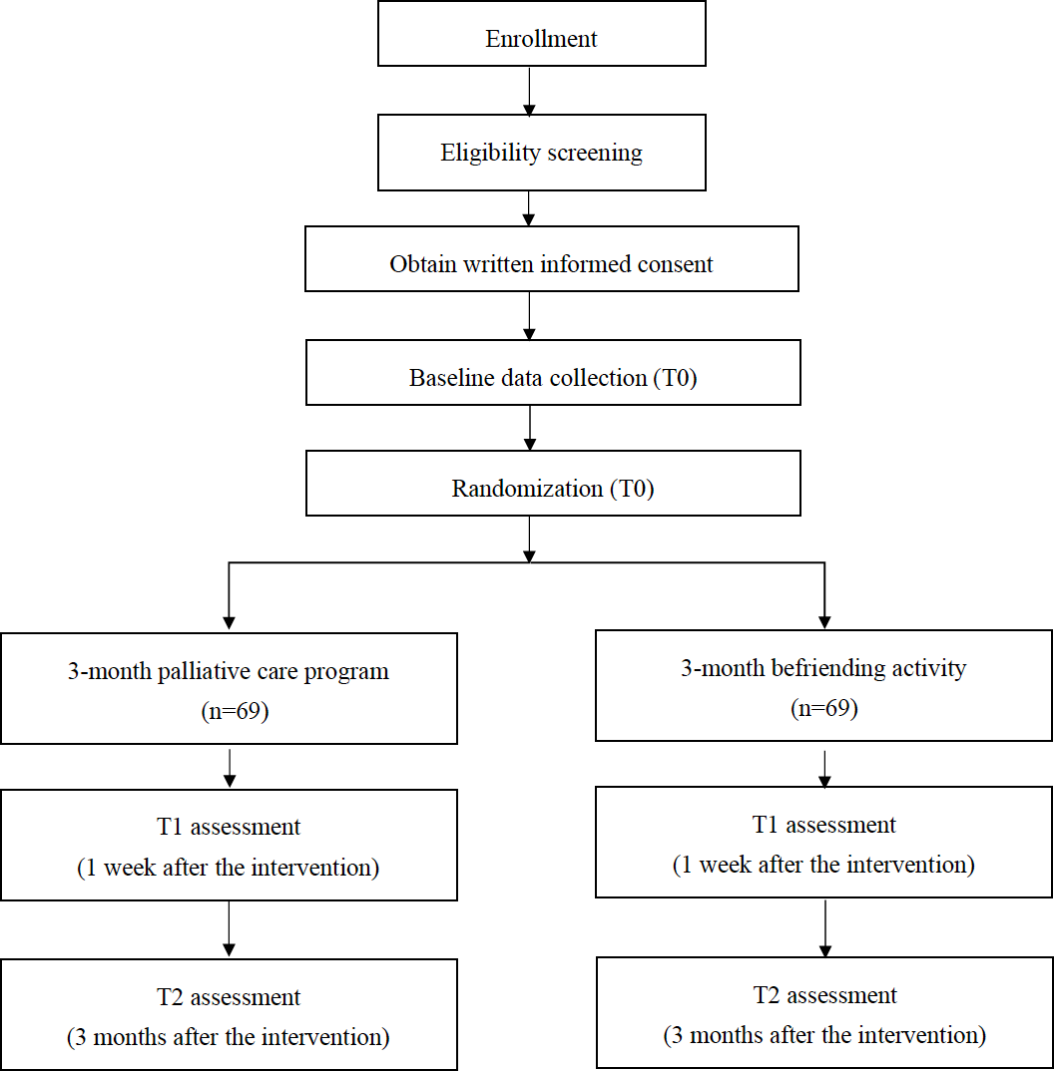
CONSORT (Consolidated Standards of Reporting Trials) flow diagram indicating the study schedule of enrollment, interventions, and assessments.

Potential participants will be provided with a general overview of the study. A research assistant will set an appointment with those patients who meet the inclusion criteria and agree to participate in the study. In the face-to-face interview, a research nurse will check the eligibility criteria and perform the baseline evaluation (T0). Information on all screened patients and reasons for exclusion will be recorded. Participants will receive a cash reward of HKD 100 (approximately US $13) per each assessment (T0, T1, and T2). To control for participant expectancy, both groups will be introduced with identical positive phrasing regarding the potential benefits of the interventions. After baseline assessment, participants will be randomly assigned to the 2 groups on a 1:1 basis using the method of permuted block with random block sizes of 4, 6, and 8 by an independent researcher not involved in the assessments. The allocation list will be computer generated by an independent researcher and concealed from other researchers and participants until the time of assignment. An independent research assistant blinded to the group allocation will collect the postintervention questionnaires. Figure 1 shows the flowchart of the study implementation plan.

### Data Analysis

Quantitative data will be analyzed using SPSS (version 28.0.1; IBM Corp) following the intention-to-treat principle. Descriptive statistics will summarize baseline characteristics, and group comparability will be examined using chi-square or Fisher exact tests. Linear mixed-effects models will assess intervention effects on primary and secondary outcomes, with time, group, and group-by-time interaction included as fixed effects. Between-group differences will be estimated using linear contrasts. Model assumptions will be checked using normal probability plots. Missing data will not be imputed as mixed-effects models accommodate incomplete observations [48]. A 2-sided significance level of 5% will be adopted. To account for the variations in therapist contact and attention, we will log the exact duration (in minutes) of each session for every participant. In sensitivity analysis, total contact time will be included as a covariate in the linear mixed-effects models to statistically control for the potential confounding effect of extra nurse contact.

Qualitative interviews will be transcribed verbatim and managed using NVivo (version 12; Lumivero). Two researchers will independently conduct inductive thematic analysis following a 5-step approach [49]. Codes will be organized into categories and themes to identify recurring patterns. An audit trail will enhance rigor, and discrepancies will be resolved through discussion. Qualitative findings will complement and enrich interpretation of the quantitative results.

### Patient and Public Involvement

Patients and the public were not involved in the design, conduct, reporting, or dissemination plans of this research protocol.

### Ethical Considerations

This study complies with the Declaration of Helsinki and has received approval from the Institutional Review Board of the University of Hong Kong and the Hospital Authority Hong Kong West Cluster (UW 22-727). Participants will receive detailed verbal and written information about the study and provide informed consent. Participation is voluntary, and individuals may withdraw at any time without consequences. To ensure confidentiality, participants will be assigned unique codes, and no identifying information will appear in study materials. Data will be securely stored, accessible only to the research team, and destroyed 7 years after study completion.

### Validity and Reliability

Intervention fidelity will be monitored through random review of 10% of audio-recorded sessions by an independent researcher with prior written consent from participants. Fidelity checklists adapted from the CALM Treatment Integrity Measure [21], ACT Fidelity Measure [50], and *Befriending Manual* [34] will be used. Quantitative data will be double entered for verification and analyzed using an intention-to-treat approach. All outcome measures have established reliability and validity in Chinese populations, and internal consistency (Cronbach α) will be calculated using study data. For qualitative rigor, 2 authors will verify transcripts against audio recordings. Coding will be conducted independently, with consensus reached through discussion.

### Dissemination

The findings of this trial will be disseminated to health care professionals, researchers, and the public through peer-reviewed publications in international journals and presentations at national or international scientific conferences.

## Results

Ethics approval was obtained on December 22, 2022. The study is funded by the Nethersole Institute of Continuing Holistic Health Education (NICHE) Research Grant from March 1, 2023, to March 1, 2025, and by the Health and Medical Research Fund (HMRF) from July 1, 2024, to September 30, 2026. Prospective clinical trial registration was completed on June 30, 2023, prior to the start of recruitment and data collection in September 2023. As of January 2026, 132 participants have been enrolled. No outcome data analyses have been conducted to date. The trial results are expected to be published in late 2026.

## Discussion

### Expected Findings

The progressive and unpredictable nature of PNDs contributes to significant psychospiritual distress, including depression, anxiety, existential distress, and loss of life meaning. While conventional care primarily focuses on motor symptom management within a biomedical framework [51], psychological and spiritual needs are often underaddressed. To bridge this gap, this study developed a novel nurse-led, community-based palliative intervention integrating CALM therapy and ACT to foster acceptance, meaning, and value-driven living across the illness trajectory.

Guided by the CSM [15], the intervention targets maladaptive illness perceptions shaped by the chronic, irreversible, and unpredictable course of PNDs, which often diminish perceived control and heighten helplessness. The integration of CALM therapy and ACT within this palliative intervention specifically aims to address these issues. CALM’s supportive-expressive approach encourages patients to explore illness-related concerns and existential issues, reframe negative thoughts, and sustain hope along the fluctuating illness trajectory, whereas ACT complements this by fostering psychological flexibility—the ability to accept difficult emotions, focus on the present, and engage in value-driven behaviors. Together, these approaches empower individuals with PNDs to navigate the uncertainty and distress of PNDs while maintaining a sense of control and purpose.

The intervention underscores the pivotal role of nurses in delivering holistic, person-centered psychospiritual care in community settings. With appropriate training and standardized protocols, nurses can address emotional and existential challenges beyond physical symptom management. By integrating relational skills, empathy, and expertise in neurological care, this study demonstrates how nursing practice can extend beyond traditional hospital-based roles to include psychotherapeutic interventions that foster psychospiritual well-being. Designed to accommodate the fluctuating course of PNDs [11], the program offers flexible delivery options, including home- or site-based sessions within a 3-month period. Individual sessions provide a private, personalized space for discussing sensitive concerns and align with evidence supporting psychological approaches for psychospiritual distress in palliative populations [52,53].

This study has important theoretical and clinical implications. Theoretically, it advances understanding of how illness perceptions influence coping and psychological outcomes in PNDs within the CSM framework and extends the application of CALM therapy and ACT to chronic progressive conditions. Clinically, it demonstrates the feasibility of nurse-led psychotherapeutic interventions in community palliative care. If effective, the program could offer a scalable model for integrating psychospiritual care into routine services. Insights from participants’ experiences will further refine the intervention’s contextual relevance and support the development of innovative, evidence-based practices in psychospiritual care for individuals with PNDs.

### Limitations

This study has several limitations. First, convenience sampling from outpatient clinics and support groups may introduce selection bias and limit generalizability to less socially engaged individuals. Second, there is a risk of contamination if control group participants become aware of the intervention content. To minimize this risk, intervention group participants will be instructed not to discuss or share any aspects of the intervention content or materials with others. Third, due to the nature of the intervention, blinding of participants is not possible. Awareness of group assignments may influence participant responses, compliance, and retention rates [54]. Fourth, the discrepancy in session duration between the intervention group and the befriending control group may introduce a confounding effect related to extra nurse contact and attention. For pragmatic reasons, the befriending control is restricted to neutral, nonaffective topics lasting 30 to 60 minutes to sustain engagement, whereas CALM therapy involves a highly semistructured 90-minute framework with interactive experiential exercises. Consequently, while the active control accounts for therapist warmth and alliance, we cannot fully separate content-specific benefits from differences in total contact time. This remains a potential bias that we will attempt to mitigate by controlling for total contact time in our sensitivity analysis. To enhance adherence, participants who complete assessments will receive a modest incentive (HKD 100 [approximately US $13] per assessment).

### Conclusions

This study addresses the significant psychospiritual distress among individuals with PNDs by developing and evaluating a nurse-led supportive-expressive palliative program integrated with acceptance and commitment–based strategies. This novel approach aims to improve the psychospiritual well-being of individuals with PNDs, enabling them to navigate illness-related distress; foster acceptance; and engage in value-driven, meaningful lives despite the challenges of PNDs. The use of a mixed methods design enhances the interpretation of quantitative findings and provides valuable insights to inform future implementation in health care services.

## Supplementary material

10.2196/105727 Checklist 1 SPIRIT 2025 checklist.

10.2196/105727 Peer Review Report 1 Peer Review Report from the Nethersole Institute of Continuing Holistic Health Education (NICHE) Research Grant 2022/2023 and the Health and Medical research Fund (HMRF 2022), Health Bureau.

## Abbreviations

ACT: acceptance and commitment therapy
CALM: Managing Cancer and Living Meaningfully
CSM: Common-Sense Model of Self-Regulation
HMRF: Health and Medical Research Fund
MS: multiple sclerosis
NICHE: Nethersole Institute of Continuing Holistic Health Education
PD: Parkinson disease
PND: progressive neurological disease
QoL: quality of life
SPIRIT: Standard Protocol Items: Recommendations for Interventional Trials
