# Supplementary material for: Effects of a Nurse-Led, Acceptance and Commitment–Based Palliative Intervention on the Psychospiritual Well-Being of Patients With Progressive Neurological Diseases: Protocol for a Mixed Methods Study
Source: JMIR Res Protoc. 2026 Aug 13;15:e105727. doi: 10.2196/105727 (PMC13472230; doi:10.2196/105727)
Supplement: Supplementary file 1 [file resprot-v15-e105727-s002.pdf]

Research Council

Grant Review Board (GRB)

GRB ASSESSMENT REPORT – GRANT APPLICATION

Application No. : 21223021

Project Title : Effects of a nurse-led palliative care programme, ‘Cultivating Acceptance and Living Meaningfully with Progressive Neurological Diseases’ (CALM-Neuro): A feasibility randomised control trial

Principal Applicant (PA) : Dr KWOK Jojo Yan-yan

Rating : 4. Recommended for support

GRB's comments

1. It is unclear how the proposed intervention, normally used for cancer patients, will be applicable to the palliative care of neurological patients. Clarify the linkage between cancer patients and patients with progressive neurological diseases.

2. Review the duration of befriending activity for the control group and elaborate on measures to avoid high dropout rate.

3. Provide justification for the budget item “Incentives for subjects” ($24,000), i.e. $300 per participant. Incentives/tokens of appreciation for study participants are usually gifts or coupons ranging from $20 to $200, depending on the level of participation.

4. Provide details and justifications for the following budget items: "License fees for questionnaires" ($4,000), “Transcription and translation fess of audiotaped interviews” ($40,000). Please also provide supporting documents (e.g. quotation) for “transcription and translation fees of audiotaped interviews”.

5. Staff cost should be based on the salary as at the date of application submitted. No salary increase over the grant period should be proposed in the budget.

6. The submitted human research ethics approval has a different project title from your HMRF application. Please provide supporting document from IRB to confirm such approval is applicable to and cover this HMRF application. Otherwise, a new approval is required.

Reviewers’ comments

- The Reviewers' comments are attached for information only.

Research Council

Grant Review Board (GRB)

Action(s) required by PA

- Refer to the checklist at https://rfs.healthbureau.gov.hk/images/HMRF/Checklist_for_fundable_projects.pdf for details in preparing your response.
- Respond to the GRB' s comments point-by-point and refer to Reviewers' comments for details.
- Submit a revised "Proposed Research Project", i.e. Section 13 of Application Form, in pdf files (both "track change" version and clean copy are required).
- Submit a revised budget plan under Sections 10a to 10d of the application form.
- Submit all the materials required via eGMS by 18 October 2023.
- Submit (a) regulatory/ethics approval(s)/evidence for accessing third-party data (all documents need to bear same project title as in the application), if applicable; (b) personal email address; and (c) mobile number via eGMS no later than 20 December 2023.

Grading for applications	Meaning
**4**<br>**Recommended for support**	Nil or very minor issues to address only
**3**<br>**Recommended for support subject to clarifications / amendments**	Minor revision and clarification required for a successful delivery
**2**<br>**Not recommended for support at present**	Major revision required for significant improvement
**1**<br>**Not supported**	Minimal impact on research/flaw in methodology/incomplete application/ out of scope of the fund

REFEREE'S ASSESSMENT FORM

Reference No.: 21223021

Project Title: Effects of a nurse-led palliative care programme, 'Cultivating Acceptance and Living Meaningfully with Progressive Neurological Diseases' (CALM-Neuro): A feasibility randomised control trial

PART A: REFEREE'S DETAILED REMARKS ON THE INDIVIDUAL SECTIONS OF THE GRANT APPLICATION

1. **Originality and Impact** What is the importance of the proposed research in terms of its originality and potential impact in the area under study? How will the research findings benefit patients and/or the healthcare system? Will the research findings improve patient care, population health, influence clinical practice and/or health services management, or inform health policy in Hong Kong and elsewhere? Have the potential facilitators and barriers to this impact being achieved been identified?

This is a very important proposed endeavor, extending interventional services to a population with progressive neurological diseases. Specifically, the researchers propose a feasibility study consisting of a randomized control trial. Recognizing that with PMD experience unmet needs, psychosocial stressors, and fluctuating trajectories in the early and middle stages of illness, they aim to expand upon the effective CALM program, already investigated in oncology populations. In an innovative fashion, they will integrate CALM and ACT to target both practical and existential needs within this population. This phase tests the feasibility, acceptance and effects of the intervention. The investigators have a thoughtful integration within clinics, including neurological clinics. They also have considered contingencies.

2. **Research Questions, Aims and Hypotheses** How specific, clearly expressed and realistic are the research questions, aims and hypotheses?

The research questions are clearly listed and feasible.
The Aim is to assess the feasibility, acceptance and effects of CALM-Neuro. They will compare the effects of the interventions on participants as compared to those in the control arm (befriending activity).

They are researching feasibility, gains in psychological distress, symptom burdens, flexibility, distress, spiritual well-being, and meaning. They also are researching level of acceptance and participant experiences.

The investigators hypothesize the CALM-Neuro is feasible and that interventional participants will derive greater benefits in the constructs targeted.

3. **Subjects and Study Methodology** (i) Is the proposed design and methodology appropriate for the study? (ii) Are sample sizes clear, justified, adequate and realistic? (iii) Are any preliminary data available? (iv) How feasible is the proposed timeframe? (v) Please also provide comments on the following (where applicable):

- For proposals submitted under Advanced Medical Research (refer to Area of Project on page 1 of application), is this a clinical study which applies advanced technologies including but not limited to biotechnology in medicine, use of drugs and treatments, clinical trials, virtual health such as telemedicine, etc., to facilitate the translation of knowledge generated from health and health services or infectious diseases studies into clinical practice and to inform health policy?
- For proposals addressing thematic priorities under Implementation Science (refer to Section 5b of the application), are the appropriate framework(s) / model(s) with the pre-set criteria proposed to evaluate/assess the barriers and facilitators of implementation outcomes clearly stated?
- For Seed Grant proposals (i.e. grant ceiling is HK$500,000), is the prospect that a successful outcome will enable scale-up to a larger project/trial and/or enhance the efficacy/effectiveness of existing practice clearly stated and feasible?

(i) the proposed design is appropriate, consisting of a feasibility randomized controlled trial. They will recruit from rehab and neurology clinics and support groups. The nature of the intervention is delineated well, as is the active control intervention (Befriending).
(II) Sample sizes are clear. For the feasibility phase, they will recruit 30 individuals to each arm. At the 6-month follow-up, they will conduct semi-structured interviews with 20 individuals from each intervention group.
(III) Preliminary data are available. The investigators recently published a cross-regional survey examining the PC needs of 186 PND patients from Hong Kong, Taiwan and Thailand. Further, in ongoing work, they researched the lived experience and care needs of PND patients with qualitative interviews as well. All of this research lays the groundwork for the proposed study.
(iv) timeline is feasible given how well integrated they are into existing networks from which they will recruit. They also clearly list criteria to scale-up to a larger project

4. **Outcomes and Data Analysis** (i) Are the primary and secondary outcomes clearly defined? (ii) Have potential problems been anticipated and addressed? (iii) Is the statistical/analytical design appropriate and clearly explained?

(I) primary and secondary outcomes are clearly listed. Primary outcomes pertain to feasibility, acceptability, satisfaction. Secondary deal with the numerous constructs they will be assessing at T0, T1, and T2.

(II) yes- contingencies are listed and considered, not limited to when to refer if individuals are emotionally distressed beyond an expected threshold, difficulty with recruitment, and the need to switch to virtual modalities.
(III)statistical design is appropriate and explained.

5. **Research Capability** Comment on (i) the research team's expertise and track record (incl. principal investigator / project team members / collaborators) and (ii) the existing facilities of the Institution where the research will be conducted.

(I) the team has conducted preliminary research in this area, positioning them to be experts in PND psychosocial needs and associated interventions. It is a strong multi-disciplinary team. Further, the CALM intervention has strong supportive data in oncology populations. This would consist of applying this intervention to this population and adding to this framework with components of ACT.
(II)facilities are adequate

6. **Budget** Is the request for research personnel, consumables, equipment and overall budget justified and reasonable? [For reference, 1 USD is equivalent to approximately 7.8 HKD]

Adequate-

7. **Ethical and Safety Considerations** Is the proposed research ethically sound? Outline any safety or ethical issues that from the proposed research and comment on whether these have been adequately addressed in the proposal. Has ethical approval been sought?

These considerations are included, not limited to when and where to refer patients experiencing heightened emotional distress. Should illness or exposure based concerns impede in-person meetings, virtual meetings are an option as well.

8. **Overall Comments and Conclusion** It is always helpful for applicants to receive constructive feedback from reviewers. What are the specific strengths and weaknesses of this proposal? Please include a brief overall appraisal of the proposal focusing on any areas for improvement and the basis for your comments, e.g. awareness of other work in the field.

Strengths:

This proposal aims to provide interventions to those with progressive neurological diseases, recognizing the fluctuating impacts on psychosocial well-being, functional status, social functioning and overall quality of life. They are building on the CALM intervention that has been very effective with oncology patients, creating a CALM+ACT intervention bundled in 90 minute sessions. They plan to assess impacts on numerous important constructs of interest over three time periods, which will be instrumental for those working with patients experiencing the uncertainty of neurological diseases. Further, their work will have implications for other disease groups. The semi-structured interviews will be very helpful in determining satisfaction and acceptance from the patient perspective. They are well integrated into existing clinics. Further, they are well versed in this area of research. Most importantly, this is an area that has unmet psychosocial needs. This important work could make a huge difference in the quality of life for those experiencing PND as well as their families and children.

Weaknesses:

I do not note weaknesses but rather some areas that could be added and/or clarified to strengthen the application.
1. There are some typographical errors in the application (e.g. p. 1 "...it is surprisingly to note...")
2. There is a missed opportunity to mention hereditary components and how many neurological conditions run through families, creating family system stressors. This, in turn, is important for children, reproductive decision making and more.
3. I have some questions about the exclusion criteria. I understand why they are listed how they are but I also wonder if you are excluding some of the healthier, wellness-oriented individuals. Many individuals (think those with hereditary MD for instance) are referred for therapy. Do you really want to exclude them or could you perform a baseline survey to ensure you are accounting for baseline variables of significance? Similarly, in our center, one provider provides elements of an ACT based intervention with all individuals. Do they recognize that this is "ACT" formally... I do not know. Should it exclude them? Worth considering

REFEREE'S ASSESSMENT FORM

Reference No.: 21223021

Project Title: Effects of a nurse-led palliative care programme, 'Cultivating Acceptance and Living Meaningfully with Progressive Neurological Diseases' (CALM-Neuro): A feasibility randomised control trial

PART A: REFEREE'S DETAILED REMARKS ON THE INDIVIDUAL SECTIONS OF THE GRANT APPLICATION

1. **Originality and Impact** What is the importance of the proposed research in terms of its originality and potential impact in the area under study? How will the research findings benefit patients and/or the healthcare system? Will the research findings improve patient care, population health, influence clinical practice and/or health services management, or inform health policy in Hong Kong and elsewhere? Have the potential facilitators and barriers to this impact being achieved been identified?

The unmet palliative care needs of patients with progressive neurological diseases (PND) are identified especially in psychospiritual care. The investigators proposed to use Managing Cancer & Living Meaningfully (CALM) therapy on PND patients because of its spiritual, existential and meaning-centered focuses. In addition, to promote flexibility and enhance copying, Acceptance and Commitment Therapy (ACT) is added. Though the two named therapies are normally used in cancer patients, with the similarity of palliative care on both types of patients, the investigators proposed to use CALM and ACT on PND patients which is rather innovative.

This study also focused on a relatively neglected area, the palliative needs of PND patients. The result of this study will shade light on policy and/or care improvement in PND patients.

2. **Research Questions, Aims and Hypotheses** How specific, clearly expressed and realistic are the research questions, aims and hypotheses?

The aim of the study is clearly stated. The study hypotheses and research questions appropriately presented echoing the objectives.

3. **Subjects and Study Methodology** (i) Is the proposed design and methodology appropriate for the study? (ii) Are sample sizes clear, justified, adequate and realistic? (iii) Are any preliminary data available? (iv) How feasible is the proposed timeframe? (v) Please also provide comments on the following (where applicable):

- For proposals submitted under Advanced Medical Research (refer to Area of Project on page 1 of application), is this a clinical study which applies advanced technologies including but not limited to biotechnology in medicine, use of drugs and treatments, clinical trials, virtual health such as telemedicine, etc., to facilitate the translation of knowledge generated from health and health services or infectious diseases studies into clinical practice and to inform health policy?
- For proposals addressing thematic priorities under Implementation Science (refer to Section 5b of the application), are the appropriate framework(s) / model(s) with the pre-set criteria proposed to evaluate/assess the barriers and facilitators of implementation outcomes clearly stated?
- For Seed Grant proposals (i.e. grant ceiling is HK$500,000), is the prospect that a successful outcome will enable scale-up to a larger project/trial and/or enhance the efficacy/effectiveness of existing practice clearly stated and feasible?

1) The inclusion and exclusion criteria are well developed. Recruitment strategies with the named potential sites are appropriate. However, the initial screening, obtaining consent and baseline data collection procedures are missing.
2) The study interventions are developed basing on previous studies with justifications. The argument of using both CALM and ACT are presented. The study interventions are described clearly. However, the Befriending sessions for the control group with 90minutes x 5 might be a bit too long. The boring sessions might cause high dropout rate in the control group. For attention control, it might not necessarily be the same duration.
3) A qualitative approach will be used for process evaluation. The sampling method and the five-step thematic analysis are discussed.

4. **Outcomes and Data Analysis** (i) Are the primary and secondary outcomes clearly defined? (ii) Have potential problems been anticipated and addressed? (iii) Is the statistical/analytical design appropriate and clearly explained?

Primary outcomes and secondary outcomes are discussed in detail in the proposal. Data processing and analysis are appropriately presented.

5. **Research Capability** Comment on (i) the research team's expertise and track record (incl. principal investigator / project team members / collaborators) and (ii) the existing facilities of the Institution where the research will be conducted.

A team with rich research experience in study field and in quantitative and qualitative methodologies.

6. Budget Is the request for research personnel, consumables, equipment and overall budget justified and reasonable? [For reference, 1 USD is equivalent to approximately 7.8 HKD]

With the complexity of the study interventions and the extensiveness in outcome evaluations, the budget is reasonable.

7. Ethical and Safety Considerations Is the proposed research ethically sound? Outline any safety or ethical issues that from the proposed research and comment on whether these have been adequately addressed in the proposal. Has ethical approval been sought?

no concern

8. Overall Comments and Conclusion It is always helpful for applicants to receive constructive feedback from reviewers. What are the specific strengths and weaknesses of this proposal? Please include a brief overall appraisal of the proposal focusing on any areas for improvement and the basis for your comments, e.g. awareness of other work in the field.

Strengths:

A well thought proposal

Weaknesses:

1) It would be better if the linkage between cancer patients and PND patients in palliative care can be further strengthened. Your argument will be more convincing.
2) the extensive attention control interventions should be revisited to avoid a high dropout rate in control as you only have 30 sample in each group.
